# *Salmonella* alters heparanase expression and reduces tumor metastasis

**DOI:** 10.7150/ijms.60281

**Published:** 2021-06-11

**Authors:** Huan-Min Chiu, Wen- Yi Chiou, Wei-Jie Hsu, Li-Hsien Wu, Ming-Hui Yang, Yu-Chang Tyan, Che-Hsin Lee

**Affiliations:** 1 Department of Orthopaedics, Kaohsiung Armed Forces General Hospital, Kaohsiung 80284, Taiwan.; 2 Department of Biological Sciences, National Sun Yat-sen University, Kaohsiung 80424, Taiwan.; 3 Aerosol Science Research Center, National Sun Yat-sen University, Kaohsiung, Taiwan, 80424, Taiwan.; 4 Department of Medical Education and Research, Kaohsiung Veterans General Hospital, Kaohsiung, Taiwan.; 5 Department of Medical Imaging and Radiological Sciences, Kaohsiung Medical University, Kaohsiung, Taiwan.; 6 Department of Medical Research, China Medical University Hospital, China Medical University, Taichung 404, Taiwan.; 7 Department of Medical Laboratory Science and Biotechnology, Kaohsiung Medical University, Kaohsiung 80708, Taiwan.; 8 Doctoral Degree Program in Marine Biotechnology, National Sun Yat-sen University, Kaohsiung 80424, Taiwan.

**Keywords:** *Salmonella*, Heparanase, tumor migration, metastasis

## Abstract

*Salmonella* causes salmonellosis, is a facultative anaerobe and is one of the common Gram-negative bacteria. *Salmonella* has anti-tumor potential and tumor-targeting activity. The heparin sulfate on cell surfaces can be cleaved by heparanase that is an endo-β-D-glucuronidase. Heparanase can destroy the extracellular matrix and is involved in tumor metastasis and angiogenic activity. Previously, *Salmonella* was demonstrated to inhibit tumor metastasis. It remains unclear whether *Salmonella* inhibits metastasis by regulating heparanase. The expression of heparanase in *Salmonella*-treated tumor cells was found to be decreased. Transwell and wound-healing assays demonstrated the inhibition of cell migration after *Salmonella* treatment. *Salmonella* was found to influence the levels of phosphate-protein kinase B (P-AKT) and phosphate-extracellular regulated protein kinases (P-ERK), which are involved in heparanase expression. *Salmonella* reduced the heparanase expression induced upregulating PERK and PAKT signaling pathways. The mice bearing an experimental metastasis tumor model was used to evaluate the anti-tumor metastatic effects of *Salmonella*. Compared with the control group, *Salmonella* significantly reduced the number of metastatic nodules and enhanced survival. The results of our study indicate that* Salmonella* plays a vital role in the inhibition of tumor metastasis through the downregulation of heparanase.

## Introduction

Tumor metastasis causes the death of cancer patients, and the complex physiological responses involved are poorly understood. Several genes and changes in proteins are participated in the process of local invasion of tumor cells 1. In a wide range of tumors, heparin sulfate can be modified by heparanase. The heparanase in tumors correlates with tumor size, metastasis, and prognosis 1, 2. The reduced tumor growth and metastasis in mice were observed in heparanase knockdown mice 3. Moreover, heparanase upregulates the expression of many growth factors that are required for metastasis, including fibroblast growth factors (FGF), vascular endothelial growth factor (VEGF), and matrix metalloproteinases (MMPs) 4-6. Heparanase is involved in tumor metastasis. Many studies have demonstrated that the prognosis of cancer patients is closely related to heparanase expression 7. Therefore, knockdown of heparanase expression in the tumor microenvironment may control the growth of tumor cells and even prolong the survival of the cancer patients 8. The use of bacteria as an anti-tumor agent dates back to the end of the 19^th^ century, and different bacteria strains have an anti-tumor effect 9. Some anaerobes can accumulate target hypoxic/necrotic areas of solid tumors and show significant anti-tumor activity. *Salmonella*, a facultative anaerobe that is capable of growing in hypoxic region of tumors, has been identified as an anti-tumor agent 10. As *Salmonella* survives and replicates under both aerobic and hypoxic conditions, it can target small metastatic lesions and more extensive tumor 11, 12. Our previous studies showed that *Salmonella* can colonize small tumor nodules and enhance the survival of lung metastatic tumor mice 13. Moreover, the expression of MMP-9 in tumors was reduced after *Salmonella* treatment 8. In the present study, *Salmonella* was found to reduce the metastasis via the inhibition of heparanase expression.

## Materials and Methods

### Cells, bacteria, plasmids, reagents and animals

The Dulbecco's Modified Eagle's Medium (DMEM) containing 10% fetal bovine serum gentamicin (50 μg/mL) was used to maintain Murine melanoma cells (B16F10) and murine breast cancer cells (4T1). A vaccine strain of* Salmonella choleraesuis* [S. *choleraesuis* subsp. *choleraesuis* (Smith) Weldin serovar Dublin (ATCC 15480)] was obtained from Bioresources Collection and Research Center (Hsinchu, Taiwan) 13. As described above, a constitutively active AKT plasmid were used in this study 14-16. The resveratrol and 4',6-Diamidino-2-Phenylindole (DAPI) a were purchased from Sigma-Aldrich (Sigma Aldrich, St. Louis, MO, USA). The National Laboratory Animal Center of Taiwan provided C57BL/6 and BABL/c mice. The Laboratory Animal Care and Use Committee of the National Sun Yat-sen-University approved the experimental protocol (permit number: 10634).

### Wound-healing and Transwell Assays

The wound-healing according to the manufacturer's instructions (IBIDI, Martinsried, Germany). The moving distance was detected after 24 h by using a microscope. The migration distances of untreated cells were set to 100% and were compared with cells treated with *Salmonella*. The cell migration according to the manufacturer's instructions (Transwell cultures (ThermoFisher Scientific, Waltham, MA, USA). Cells were stained with DAPI and counted under a fluorescence microscope 8.

### Western Blotting and Transfection

The protein content was determined by a bicinchoninic acid (BCA) protein assay (Pierce Biotechnology, Rockford, IL, USA). The protein samples was fractionated by SDS-PAGE. Meanwhile, the hybond-enhanced chemiluminescence nitrocellulose membranes (Pall Life Science, Glen Cove, NY, USA) was used to transfer the protein samples. The membranes were incubated with various antibodies, including heparanase (M-45, Santa Cruz Biotechnology, Santa Cruz, CA, USA), AKT (Santa Cruz Biotechnology), phosphorylation-AKT (Santa Cruz Biotechnology), ERK (Santa Cruz Biotechnology), phosphorylation-ERK (Santa Cruz Biotechnology), and β-actin (Sigma-Aldrich). The appropriate horseradish-peroxidase-conjugated secondary antibodies were used and enhanced chemiluminescence system (T-Pro Biotechnology, New Taipei City, Taiwan) to detect the protein-antibody complexes 17-19. Lipofectamine 2000 was used to transfect with the constitutively active AKT plasmids to cells. The cells treated with resveratrol for 2 h. After post-transfection or treatment, cells were or were not treated with* Salmonella* for 90 min. The cell lysates were then harvested.

### Bacterial infection

Various tumor cells (10^5^/well) were cultured in 6 well-plates overnight. Subsequently, 0, 10^5^, 10^7^, 2 × 10^7^ (for B16F10 and 4T1 cells) colony-forming units (cfu) of *Salmonella* were added to these cells which were cultured in 1 ml of antibiotic-free medium. Tumor cells were incubated for 1.5 h at 37 °C. All the cells were washed, replenished with gentamicin (100 μg/ml)-containing complete medium, and further cultured for 16 h.

### Quantitative real-time RT-PCR

The levels of heparanase mRNA in tumor cells infected with *Salmonella* (multiplicities of infection, MOI = 1, 100, 200), or mock-infected were determined by the quantitative real-time RT-PCR. Total cellular RNA was isolated and reversed transcribed into cDNA using standard methods. PCR amplification was carried out in the LightCycler system (Roche, Mannheim, Germany) and data analyzed with LightCycler software 3.3 (Roche). The specific primer pairs used for detecting mouse heparanase and β-actin were 5'- CGA CCG ACG ACG TGG TAG AC and 5'- GCA ACA GCT CCT GGA AGG G, as well as 5'-TGG AAT CCT GTG GCA TCC ATG AAA C and 5'-TAA AAC GCA GCT CAG TAA CAG TCC G, respectively. The copy number of the heparanase gene in each sample was extrapolated from the corresponding standard curve by the indicated software and normalized with the amount of β-actin in the same sample.

### Proliferation assay

*Salmonella* at various MOIs in serum-free medium treated with cells (10^6^/well) for 1.5 h Cell Counting Kit-8 (Sigma-Aldrich, St. Louis, MO, USA) was used to detect the number of cells.

### Mouse experiments

The B16F10 (10^5^) and 4T1 cells (10^5^) admixed with or without *Salmonella* (MOI = 200) for 1.5 h and C57BL/6 and BALB/c mice were injected with *Salmonella*-treated or non-treated-cells via the tail vein on Day 0. Tumor-bearing mice were sacrificed, and the serum and lungs were removed, weighed, and histologically examined on day 20 13. The heparanase content was measured by an enzyme-linked immunosorbent assay (ELISA) kit (LifeSpan BioSciences, Inc, Seattle, WA, USA). In a parallel experiment, mice were monitored for survival.

### Statistical analysis

We determined differences between groups by using an unpaired, two-tailed Student's t-test. The Kaplan-Meier survival curve and log-rank test to measure a survival analysis. A p value less than 0.05 was considered to be statistically significant.

## Results

### *Salmonella* reduced heparanase expression* in vitro*

**Figure 1:**
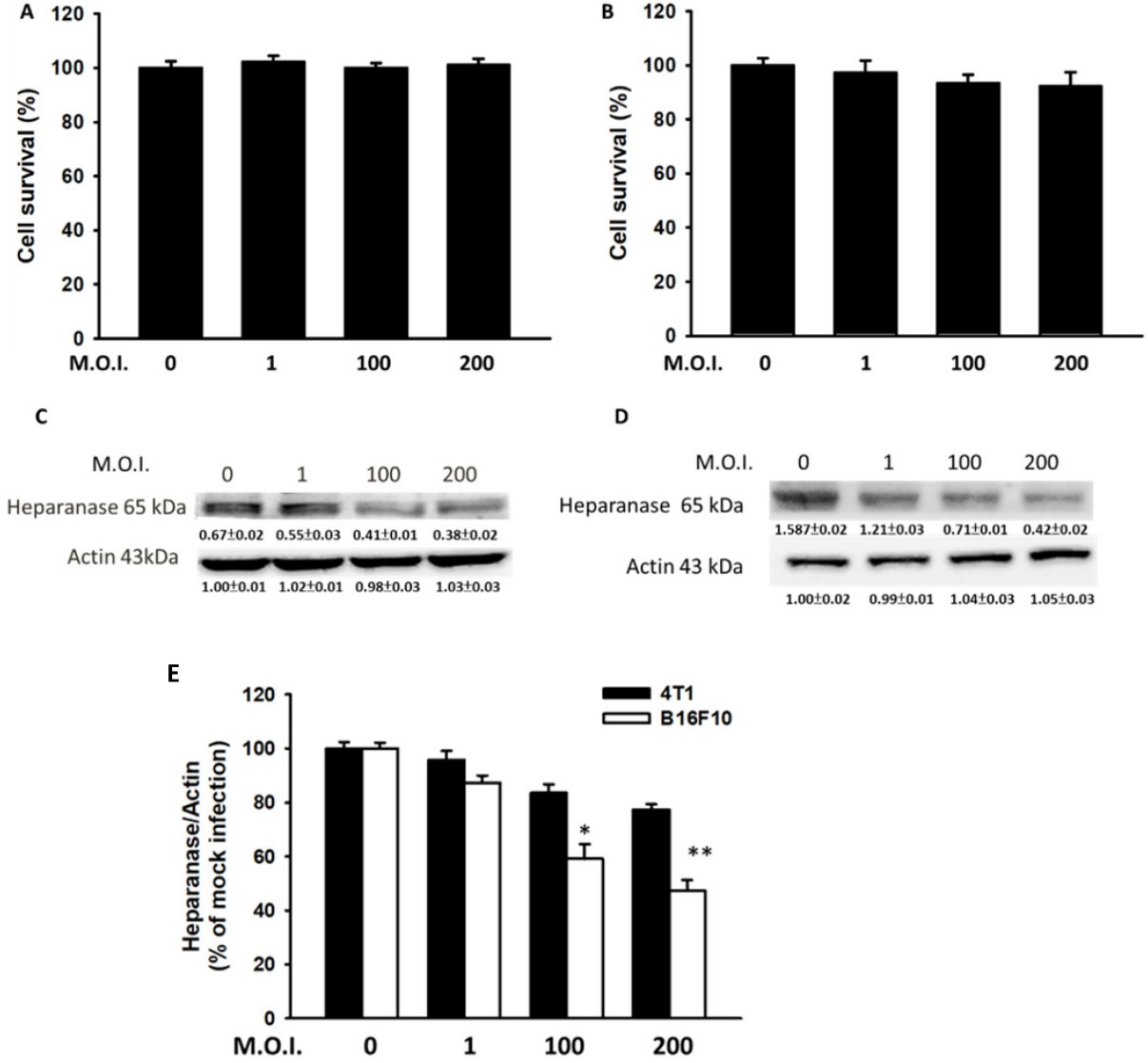
*Salmonella* (S.C.) regulated cell survival and heparanase protein levels. After infection with *Salmonella* at various multiplicities of infection (MOIs) for 1.5 h, a cell proliferation assay was used in the (A) 4T1 and (B) B16F10 cells. (n = 6, mean ± SD). The Western blotting was used to detect the protein expression in (C) 4T1 and (D) B16F10 cells. The immunoblotting assay was repeated three times with similar results. Inserted values indicated relative proteins expression in comparison with β-actin. (E) The real time RT-PCR was used to detect the mRNA expression of heparanase in 4T1and B16F10 cells.

Mouse breast cancer B16F10 melanoma and 4T1 cells were used to study the anti-migration activity of *Salmonella*. Figure 1 shows the cell survival of and heparanase expression in tumor cells after treatment with *Salmonella* at various MOIs. We found those MOIs (1-200) that did not induce cytotoxicity after infection with *Salmonella* for 90 min and used the conditions to study the heparanase production after *Salmonella* treatment (Fig. 1A and B). The expressions of heparanase in 4T1 and B16F10 cells were significantly reduced in tumor cells after infection with *Salmonella* (MOI=200) (Fig. 1C and D) (p<0.05 for S.C. MOI=0 versus MOI=200 in 4T1; p<0.01 for S.C. MOI=0 versus MOI=200 in B16F10). Treatment with an increased amount of *Salmonella* significantly downregulated the expression of heparanase in the two types of tumor cells. As shown in Fig. 1E, the levels of heparanase mRNA were reduced after *Salmonella* treatment. Herein, these studies indicate that *Salmonella* inhibits the protein expression of heparanase in tumor cells.

### *Salmonella* inhibited tumor cell migration

**Figure 2:**
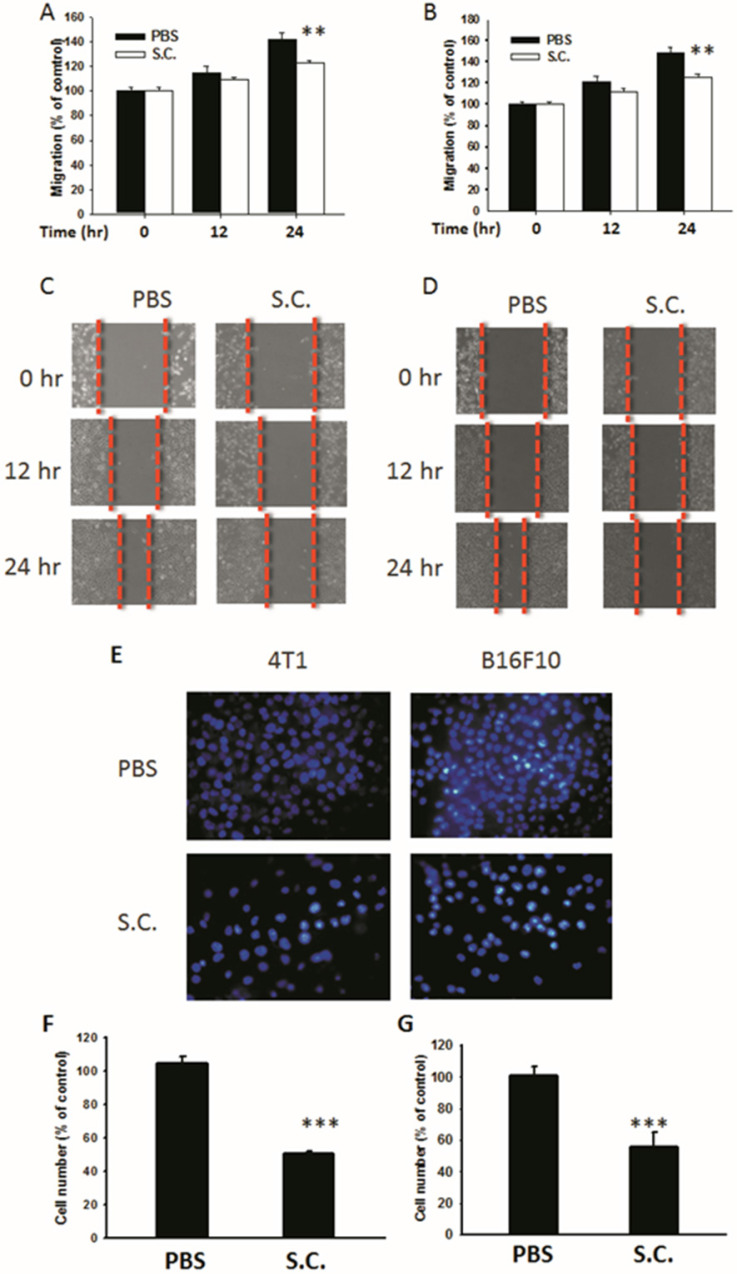
The cellular motility of 4T1 and B16F10 cells after *Salmonella* (S.C.) treatment. The cells were co-cultured with* Salmonella* (MOI = 200) for 1.5 h. The motility distances of different groups of (A) 4T1 cells and (B) B16F10 cells were measured and are shown in (C, D). The (F) 4T1 cells and (G) B16F10 cells were placed on the upper layer of Tranwell and then infected with *Salmonella* (MOI = 200) for 90 min. After 24 h, the bottom layer of cells were stained with 4',6-diamidino-2-phenylindole (DAPI) and counted under a fluorescence microscope (E) (n = 6, mean ± SD. ** p < 0.01; *** p < 0.001).

*Salmonella*-infected 4T1 mouse breast and B16F10 mouse melanoma cells were examined to determine *Salmonella*'s activity to reduce the migration of tumor cells. The results of the wound-healing assay showed that, upon the addition of *Salmonella*, the movement of 4T1 cells was inhibited compared with that of the control group (Fig. 2A). A similar phenomenon was observed in B16F10 cells infected with *Salmonella* (Fig. 2B). A wound-healing test was used to observe the reduction of the motility of *Salmonella*-treated tumor cells (Fig. 2C and D). Although *Salmonella* did not affect cell proliferation after a short period of infection, there remains the possibility of a reduction in cellular proliferation after *Salmonella* infection. The results of Transwell assay showed that the migration of 4T1 and B16F10 cells was reduced after treatment with *Salmonella* (Fig. 2E). After counting the number of migrated tumor cells, the movement of both types of tumor cells was found to be severely affected by *Salmonella* treatment (Fig. 2F and G). These results suggest that *Salmonella* reduces the motility of tumor cells.

### *Salmonella* inhibited the expression of heparanase via the phosphate-protein kinase B (P-AKT) and phosphate-extracellular regulated protein kinases (P-ERK) pathways

**Figure 3:**
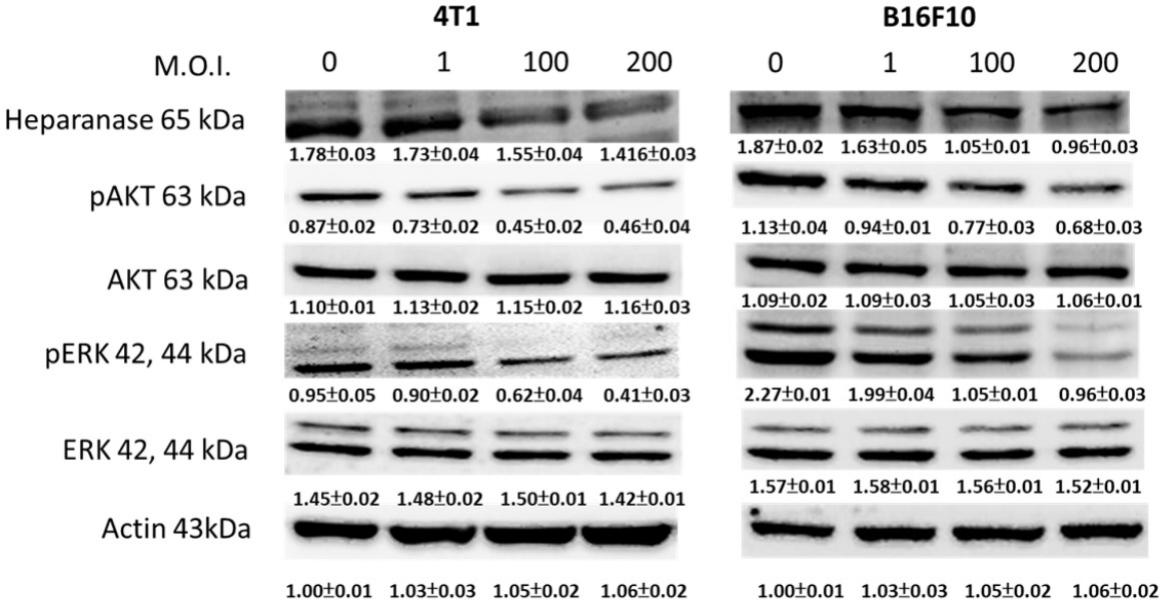
The heparanase expression in *Salmonella*-treated-4T1 and -B16F10 cells. The cells were co-cultured with* Salmonella* (MOI = 1-200) for 1.5 h. The protein expression in 4T1 and B16F10 cells was measured. The immunoblotting assay was repeated three times with similar results. Inserted values indicated relative proteins expression in comparison with β-actin.

We used Western blotting to evaluate the potential signaling pathways through which *Salmonella* has its anti-migration effects and examine the expression of heparanase and related signaling pathways (Fig. 3). Heparanase has enzyme activity and induce metastasis. The protein levels of heparanase were inhibited in tumor cells, treated with *Salmonella* including 4T1 and B16F10 cells (Fig. 3) (p<0.05 for S.C. MOI=0 versus MOI=200 in 4T1 and B16F10). This result is consistent with the results shown in Figure 1. Because* Salmonella* can influence the protein levels of heparanase in two types of tumor cells, *Salmonella* induced the potential signaling pathway in tumor cells. Some reports have found that AKT and ERK can regulate heparanase synthesis 20. Previous studies have shown that *Salmonella* can downregulate phosphate-AKT expression 21. In this study, treatment with *Salmonella* was found to reduce the AKT phosphorylation in two tumor cells (Fig. 3) (p<0.01 for S.C. MOI=0 versus MOI=200 in 4T1 and B16F10). This effect appears to occur in a dose-dependent manner. Phosphate-ERK was also found to be involved in the regulation of cellular heparanase expression 20. After infection with *Salmonella*, the expression of phosphate-ERK was significantly inhibited in both cell lines (p<0.01 for S.C. MOI=0 versus MOI=200 in 4T1 and B16F10). These results suggest that *Salmonella* inhibits heparanase expression in 4T1 and B16F10 cells through downregulation of the ERK and AKT signaling pathways.

### *Salmonella* reduced heparanase expression via inhibiting the phosphate-AKT and phosphate-ERK signaling pathways

**Figure 4:**
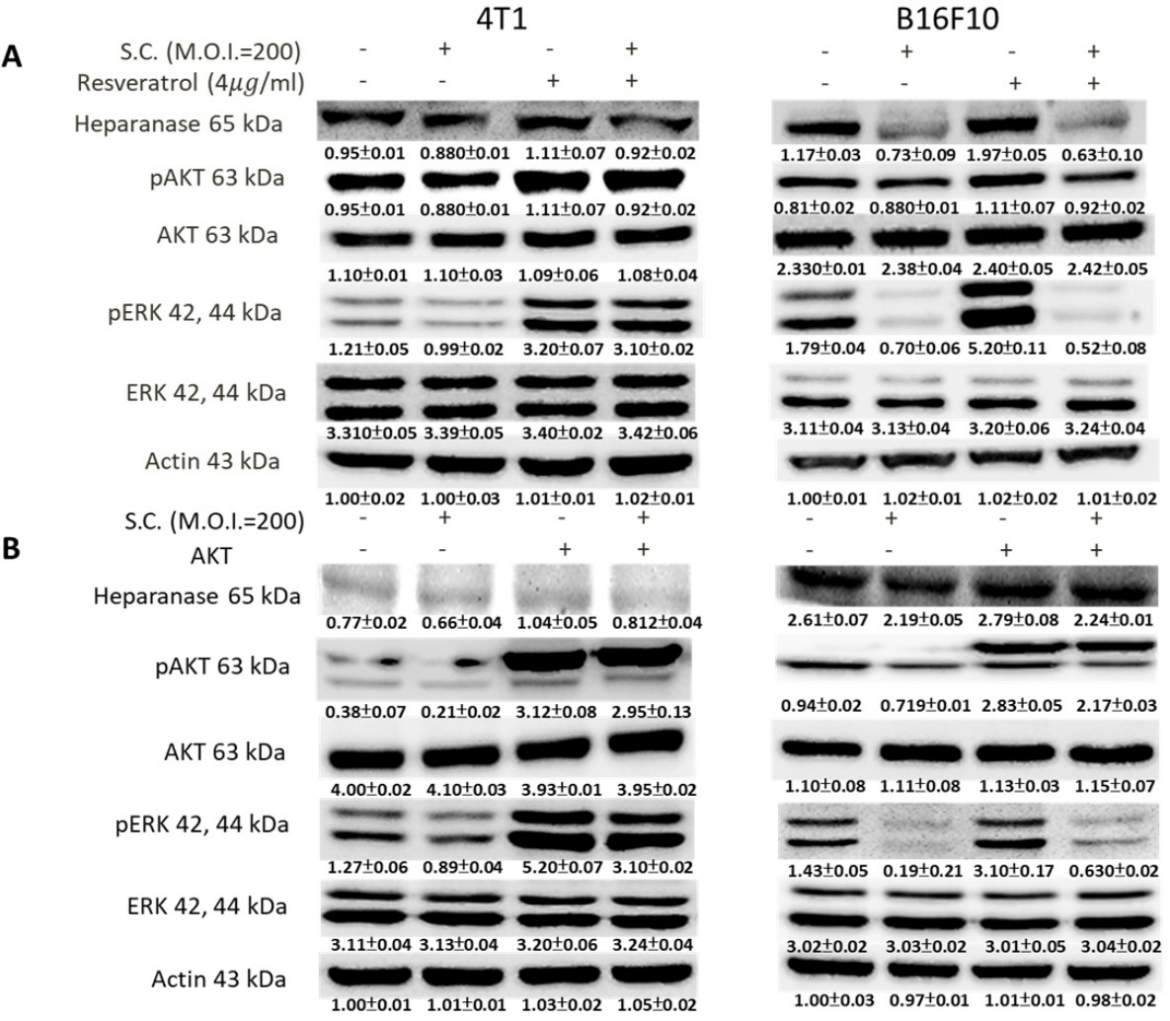
The ERK and AKT signaling pathways were participated in *Salmonella* (S.C.)-mediated HPSE expression. (A) The 4T1 and B16F10 cells were infected with *Salmonella* (MOI = 200) at the concentration of 5 µg/mL for 2 h with resveratrol. The protein expression in 4T1 and B16F10 cells was measured. (B) The 4T1 and B16F10 cells were transfected with an active AKT plasmid. The cells were treated with *Salmonella* (MOI = 200) for 1.5 h after 16 h. The various protein expressions in 4T1 and B16F10 cells was measured. The immunoblotting assay was repeated three times with similar results. Inserted values indicated relative proteins expression in comparison with β-actin.

**Figure 5:**
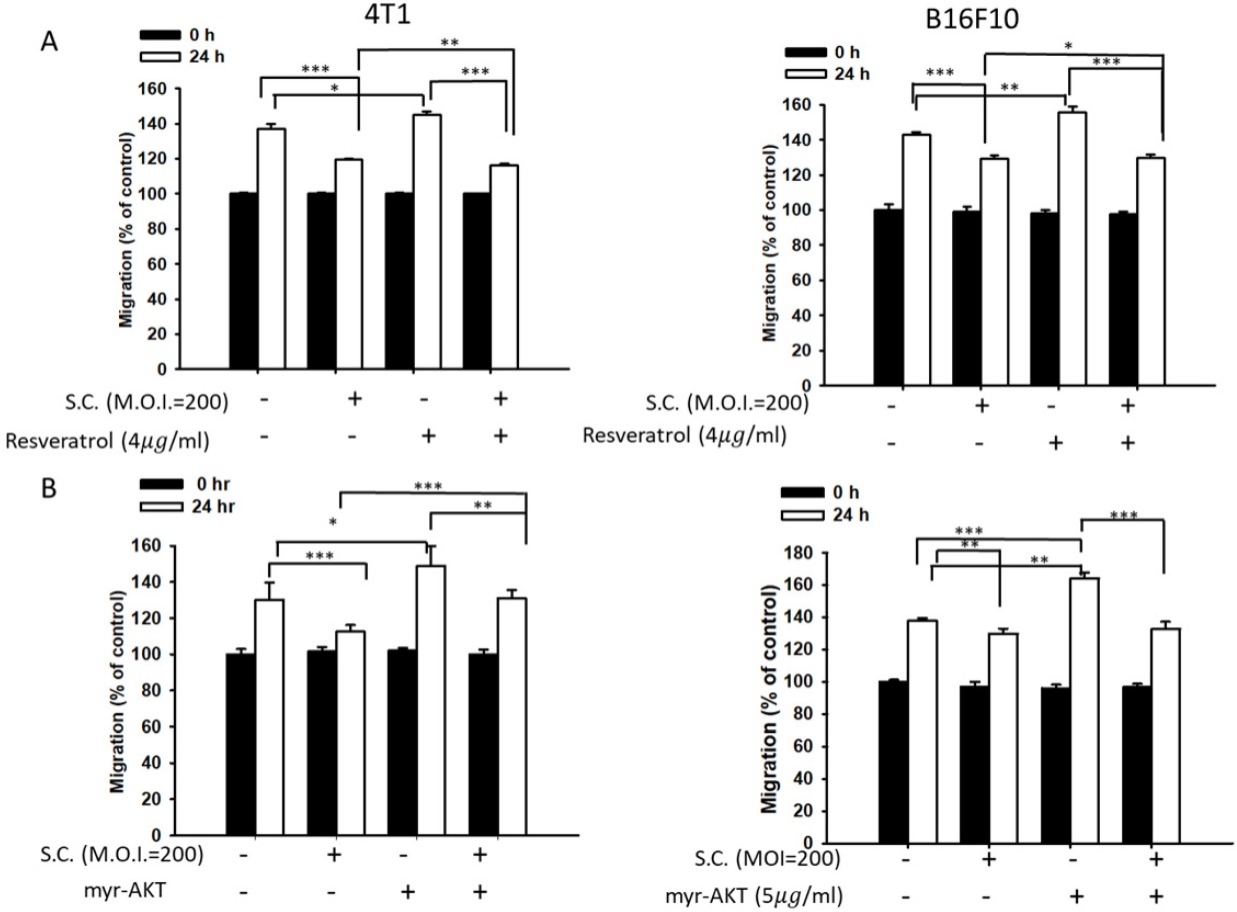
A wound-healing assay showed that the AKT and ERK signaling pathways were participated in the *Salmonella* (S.C.)-mediated inhibition of tumor cell migration. (A) The 4T1 and B16F10 cells were infected with *Salmonella* (MOI = 200) at the concentration of 5 µg/mL for 2 h with resveratrol. The moving distance of 4T1 and B16F10 cells was measured. (B) The 4T1 and B16F10 cells were transfected with active AKT plasmids. After 16 h, the cells were treated with *Salmonella* (MOI = 200) for 1.5 h. The migration distance of 4T1 and B16F10 cells was measured (n = 6, mean ± SD. * p < 0.05; ** p < 0.01; *** p < 0.001).

Phosphate-ERK plays a vital role in *Salmonella*-induced downregulation of the heparanase pathway. Moreover, resveratrol treatment can upregulate phosphate-ERK 22. When phosphate-ERK is upregulated after resveratrol treatment, heparanase expression is also increased (Fig. 4A) (p<0.01 for Mock versus resveratrol in 4T1 and B16F10). *Salmonella* can reduce the expression of heparanase in resveratrol-treated cells (p<0.05 for S.C. versus resveratrol in 4T1 and B16F10). Furthermore, *Salmonella* decreased heparanase expression in B16F10 and 4T1 tumor cells by suppressing the phosphorylation of AKT. The transfection of plasmids encoding active form of AKT can rescue the AKT signaling pathway 23. The suppressive effect of *Salmonella* on phosphate-AKT was reduced after transfecting the constitutively active form of AKT plasmids in the two types of cells (Fig. 4B) (p<0.05 for S.C. versus S.C. + AKT in 4T1 and B16F10). Transfection of plasmids encoding an active form of AKT increased the expression of heparanase after *Salmonella* treatment. After transfecting the constitutively active form of AKT, phosphate-ERK was upregulated in the two cell lines (p<0.05 for Mock versus AKT in 4T1 and B16F10). Phosphate-AKT was not influenced in the resveratrol-treated group (p>0.05 for AKT versus resveratrol in 4T1 and B16F10). These results suggest that AKT is upstream of ERK in* Salmonella*-regulated heparanase in 4T1 and B16F10 cells. As previously described, *Salmonella* significantly reduced the moving distance of tumor cells. The moving distances of *Salmonella*-treated-4T1 and -B16F10 cells could be reversed by treatment with resveratrol or transfection of a constitutively active AKT plasmid (Fig. 5). The cellular migration behavior was consistent with the results of Western blotting. These results demonstrated that the AKT/ERK signaling pathway might involve in the *Salmonella*-regulated heparanase expression and tumor cell migration behavior.

### *Salmonella* reduced tumor metastasis* in vivo*

**Figure 6:**
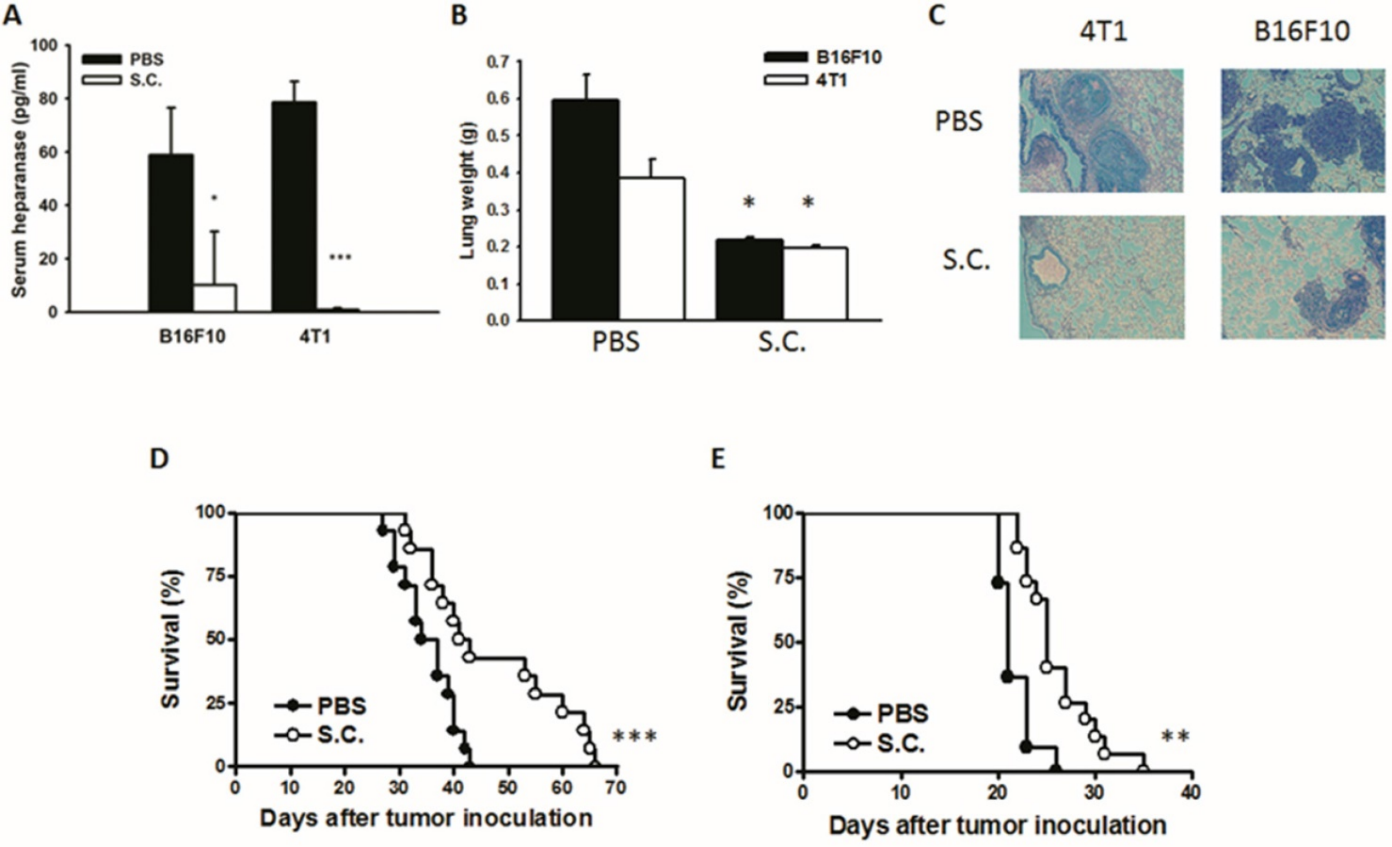
The expression of heparanase was reduced after *Salmonella* treatment *in vivo*. Mice were injected with tumor cells (10^5^) admixed with or without *Salmonella* (MOI = 200) for 1.5 h via the tail vein. At Day 20, the mice were sacrificed. (A) The serum was collected and the protein levels of HPSE were measured. (B) The anti-tumor effect of *Salmonella* was measured by lung weight (n = 4, data are expressed as mean ± SD. * p < 0.05; *** p < 0.001). (C) An example of representative is the picture of metastatic nodules 20 days after intravenous injection of 4T1 or B16F10 cells (10^5^). Kaplan-Meier survival curves of mice bearing *Salmonella*-treated (D) 4T1 and (E) B16F10 tumors are shown (n = 13-15. ** p < 0.01; *** p < 0.001).

The tumor cells can release heparanase and digest extracellular matrixes to help cell migration to distant sites. We previously established a platform that screens for anti-metastatic molecules 8, 24. The tumor cells admixed with *Salmonella* (MOI = 200) and then injected into mice. We investigated whether *Salmonella* could inhibit pulmonary tumor nodules. Mice bearing metastatic nodules were sacrificed after inoculation of the tumors for 20 days. Serum was collected and analyzed by ELISA. The heparanase in mice treated with *Salmonella* was significantly decreased in comparison with the PBS group (Fig. 6A). To quantitatively determine tumor burden, the weight of wet lung was measured. The mice injected with 4T1 and B16F10 tumor cells admixed with *Salmonella* had 63% and 48% lower wet lung weight, respectively, compared with those injected with cells admixed with PBS (Fig. 6B). The numerous pulmonary nodules were observed in the lungs from PBS-treated mice. However, the smaller and fewer tumor nodules were observed in the lungs from the mice treated with *Salmonella* (Fig. 6C). In the two metastatic tumor models, the survival of the mice treated with Salmonella was significantly enhanced (Fig. 6D and E). Collectively, these results show that *Salmonella* reduced pulmonary tumor nodule growth and prolonged the survival of mice via downregulation of heparanase expression.

## Discussion

Researchers have found *Salmonella* to possess tumor-targeting potential and considered *Salmonella* to be new strategies in the treatment of tumors 25, 26. *Salmonella* has many tumor-growth-inhibiting qualities, including gene transfer, increasing host immunity, reducing angiogenesis, and inhibiting metastasis 27, 29. The mechanism underlying the anti-tumor activity of *Salmonella* remains vague. Herein, *Salmonella* reduced the function and expression of heparanase, a major enzyme involved in tumor metastasis, in tumor cells via the AKT/ERK signaling pathway. *Salmonella*-treated groups had lower heparanase expression, a lower number of metastatic nodules, and lower lung weights in animal models compared with controls.

Heparanase not only enhances tumor metastasis but is also involved in the regulation of multiple proteins that promote the aggressive biological behavior of tumor, including VEGF and MMP-9 3-6. Previously, we found that* Salmonella* can reduce tumor migration via reducing MMP-9 8. Herein, *Salmonella* was found to potentially reduce MMP-9 via heparanase 30. Increasing evidence suggests that heparanase exerts an extreme influence on host immune cells. In transgenic mice, less neutrophil infiltration was observed 31. When* Salmonella* accumulates at a tumor site, neutrophils are attracted to tumors 13. *Salmonella*-downregulated heparanase expression in tumors may induce neutrophil infiltration. To elucidate the potential mechanism, *Salmonella* suppressed the expression of AKT/ERK and inhibited heparanase expression. Consistent with the downregulation of heparanase expression, the tumor migration behavior was inhibited. The downregulation of heparanase expression was associated with a decrease in the hypoxia-inducible factor-1α (HIF-1α) level, meaning that the expression of HIF-1α is associated with heparanase 32. *Salmonella* has previously been reported to decrease HIF-1α expression 33. Mechanistically, we found that *Salmonella* regulated tumor metastasis by downregulating heparanase expression through the AKT/ERK signaling pathway. Heparanase is a multifunctional protein that influenced metastasis and tumor growth. Therefore, heparanase is a viable target for tumor therapy and anti-heparanase is a promising anticancer agent. As a result, several anti-heparanase drugs have been developed to treat tumor 31. The common side effect associated with anti-heparanase drugs is anti-coagulant activity 34. Therefore, the ability of *Salmonella* to target multiple tumors from distant sites makes it an ideal anti-tumor agent over some other cancer therapeutic agents limited to local administration. The anti-heparanse activity was specific in tumor sites by tumor-targeting *Salmonella*.

Previously, we demonstrated that *Salmonella* as a single-agent could inhibit tumor growth and enhance survival in mice tumor models 13. The additive antitumor effects could be observed in the combination therapy of *Salmonella* plus cisplatin. Our previous findings point out that *Salmonella* in combination with cisplatin, which exerts oncolytic effects and enhances antitumor immune responses, represents a promising strategy for the treatment of primary and metastatic tumors 13.

The tumor growth rate might be critical factor for* Salmonella* treatment. The growth rate of B16F10 cells was faster than that of 4T1 cells* in vitro* and *in vivo*. The rapidly growing B16F10 cells are more sensitive to *Salmonella*-mediated response than the 4T1 cells. The AKT/ERK signal pathways in B16F10 cells are more easily influenced after *Salmonella* treatment compared with 4T1 cells (Fig. 4). The growth rate of tumor might be a critical factor involved *Salmonella* treatment. Furthermore, we have demonstrated that *Salmonella* can inhibit tumor growth in tumor-bearing mice 14, 17. We investigate the possible mechanism causing the regression. *Salmonella*-induced decrease of immune checkpoints can be only seen *in vivo* but not *in vitro*. We can verify these findings through analysis of CD4 and CD8 positive cells. However, *Salmonella* may have pleiotropic activities that can directly and indirectly affect tumor immunity processes in 4T1 tumor models *in vivo*. As shown in *Salmonella*-treated 4T1 tumor model, the slightly growth inhibition of 4T1 was observed. The phenomena might be related to* Salmonella*-mediated the downregulation of immune checkpoints.

Our results demonstrated that downregulating the protein levels of heparanase with *Salmonella* infection significantly reduced the migration of mouse tumor cells* in vitro* and *in vivo*. By analysis of the pleiotropic activities of *Salmonella*, we suggest that *Salmonella* not only inhibits primary tumor growth but also reduces metastasis.

## References

[1] Sanderson RD, Elkin M, Rapraeger AC, Vlodavsky I (2017). Heparanase regulation of cancer, autophagy and inflammation: new mechanisms and targets for therapy. FEBS J.

[2] Chen X, Jiang W, Yue C, Zhang W, Tong C, Dai D, Cheng B, Huang C, Lu L (2017). Heparanase contributes to trans-endothelial migration of hepatocellular carcinoma cells. J Cancer.

[3] Edovitsky E, Elkin M, Zcharia E, Peretz T, Vlodavsky I (2004). Heparanase gene silencing, tumor invasiveness, angiogenesis, and metastasis. J Natl Cancer Inst.

[4] Zetser A, Bashenko Y, Edovitsky E, Levy-Adam F, Vlodavsky I, Ilan N (2006). Heparanase induces vascular endothelial growth factor expression: correlation with p38 phosphorylation levels and Src activation. Cancer Res.

[5] Nasser NJ, Avivi A, Shafat I, Edovitsky E, Zcharia E, Ilan N, Vlodavsky I, Nevo E (2009). Alternatively spliced Spalax heparanase inhibits extracellular matrix degradation, tumor growth, and metastasis. Proc Natl Acad Sci USA.

[6] Cohen-Kaplan V, Naroditsky I, Zetser A, Ilan N, Vlodavsky I, Doweck I (2008). Heparanase induces VEGF C and facilitates tumor lymphangiogenesis. Int J Cancer.

[7] Purushothaman A, Sanderson RD (2020). Heparanase: A Dynamic Promoter of Myeloma Progression. Adv Exp Med Biol.

[8] Tsao YT, Kuo CY, Cheng SP, Lee CH (2018). Downregulations of AKT/mTOR signaling pathway for Salmonella-mediated suppression of matrix metalloproteinases-9 expression in mouse tumor models. Int J Mol Sci.

[9] Pawelek JM, Low KB, Bermudes D (2003). Bacteria as tumour-targeting vectors. Lancet Oncol.

[10] Pawelek JM, Low KB, Bermudes D (1997). Tumor-targeted Salmonella as a novel anticancer vector. Cancer Res.

[11] Pangilinan CR, Lee CH (2019). Salmonella-based targeted cancer therapy: updates on a promising and Innovative tumor immunotherapeutic strategy. Biomedicines.

[12] Chang WW, Lee CH (2014). Salmonella as an innovative therapeutic antitumor agent. Int J Mol Sci.

[13] Lee CH, Wu C.L, Tai YS, Shiau AL (2005). Systemic administration of attenuated Salmonella choleraesuis in combination with cisplatin for cancer therapy. Mol Ther.

[14] Chen MC, Pangilinan CR, Lee CH (2020). Salmonella Breaks Tumor Immune Tolerance by Downregulating Tumor Programmed Death-Ligand 1 Expression. Cancers.

[15] Wang WK, Chiang WC, Lai CH, Lee CH (2018). Salmonella-mediated cytolethal distending toxin transfer inhibits tumor growth. Hum Gene Ther.

[16] Wang CC, Yang CJ, Wu LH, Lin HC, Wen ZH, Lee CH (2018). Eicosapentaenoic acid reduces indoleamine 2,3-dioxygenase 1 expression in tumor cells. Int J Med Sci.

[17] Kuan YD, Lee CH (2016). Salmonella overcomes tumor immune tolerance by inhibition of tumor indoleamine 2, 3-dioxygenase 1 expression. Oncotarget.

[18] Yang CJ, Kuo CT, Wu LH, Chen MC (2019). Pangilinan CR, Phacharapiyangkul N, Liu W, Chen YH, Lee CH. Eicosapentaenoic acids enhance chemosensitivity through connexin 43 upregulation in murine melanoma models. Int J Med Sci.

[19] Chang HL, Kuo YH, Wu LH, Chang CM, Cheng KJ, Tyan YC, Lee CH (2020). The extracts of Astragalus membranaceus overcome tumor immune tolerance by inhibition of tumor programmed cell death protein ligand-1 expression. Int J Med Sci.

[20] Spyrou A, Kundu S, Haseeb L, Yu D, Olofsson T, Dredge K, Hammond E, Barash U, Vlodavsky I (2017). Forsberg-Nilsson K. Inhibition of heparanase in pediatric brain tumor cells attenuates their proliferation, invasive capacity, and *in vivo* tumor growth. Mol Cancer Ther.

[21] Lee CH, Lin ST, Liu JJ, Chang WW, Hsieh JL (2014). Wang WK. Salmonella induce autophagy in melanoma by the downregulation of AKT/mTOR pathway. Gene Ther.

[22] Cheng YJ, Chang MY, Chang WW, Wang WK, Liu CF, Lin ST, Lee CH (2015). Resveratrol enhances chemosensitivity in mouse melanoma model through connexin 43 upregulation. Environ Toxicol.

[23] Yang CJ, Chang WW, Lin ST, Chen MC, Lee CH (2018). Salmonella overcomes drug resistance in tumor through p-glycoprotein downregulation. Int J Med Sci.

[24] Chen MC, Chang WW, Kuan YD, Lin ST, Hsu HC, Lee CH (2012). Resveratrol inhibits LPS-induced epithelial-mesenchymal transition in mouse melanoma model. Innate Immun.

[25] Forbes NS (2010). Engineering the perfect (bacterial) cancer therapy. Nat Rev Cancer.

[26] Zhao M, Yang M, Li XM, Jiang P, Baranov E, Li S, Xu M, Penman S, Hoffman RM (2005). Tumor-targeting bacterial therapy with amino acid auxotrophs of GFP-expressing Salmonella typhimurium. Proc Natl Acad Sci USA.

[27] Hiroshima Y, Zhang Y, Zhao M, Zhang N, Murakami T, Maawy A, Mii S, Uehara F, Yamamoto M, Miwa S, Yano S, Momiyama M, Mori R, Matsuyama R, Chishima T, Tanaka K, Ichikawa Y, Bouvet M, Endo I, Hoffman RM (2015). Tumor-targeting Salmonella typhimurium A1-R in combination with Trastuzumab eradicates HER-2-positive cervical cancer cells in patient-derived mouse models. PLoS One.

[28] Yam C, Zhao M, Hayashi K, Ma H, Kishimoto H, McElroy M, Bouvet M, Hoffman RM (2010). Monotherapy with a tumor-targeting mutant of S. typhimurium inhibits liver metastasis in a mouse model of pancreatic cancer. J Surg Res.

[29] Lee CH (2012). Engineering bacteria toward tumor targeting for cancer treatment: current state and perspectives. Appl Microbiol Biotechnol.

[30] Purushothaman A, Chen L, Yang Y, Sanderson RD (2008). Heparanase stimulation of protease expression implicates it as a master regulator of the aggressive tumor phenotype in myeloma. J Biol Chem.

[31] Escobar Galvis ML, Jia J, Zhang X, Jastrebova N, Spillmann D, Gottfridsson E, van Kuppevelt TH, Zcharia E, Vlodavsky I, Lindahl U, Li JP (2007). Transgenic or tumor-induced expression of heparanase upregulates sulfation of heparan sulfate. Nat Chem Biol.

[32] Z CZ, Luo C, Yang Z, Wang L (2010). Heparanase participates in the growth and invasion of human U-2OS osteosarcoma cells and its close relationship with hypoxia-inducible factor-1α in osteosarcoma. Neoplasma.

[33] Tu DG, Chang WW, Lin ST, Kuo CY, Tsao YT, Lee CH (2016). Salmonella inhibits tumor angiogenesis by downregulation of vascular endothelial growth factor. Oncotarget.

[34] Groult H, Cousin R, Chot-Plassot C, Maura M, Bridiau N, Piot JM, Maugard T, Fruitier-Arnaudin I (2019). λ-Carrageenan oligosaccharides of distinct anti-heparanase and anticoagulant activities inhibit MDA-MB-231 breast cancer cell migration. Mar Drugs.

